# Preexisting radiological interstitial lung abnormalities are a risk factor for severe radiation pneumonitis in patients with small-cell lung cancer after thoracic radiation therapy

**DOI:** 10.1186/s13014-018-1030-1

**Published:** 2018-05-02

**Authors:** Fangjuan Li, Ziyang Zhou, Ailu Wu, Yong Cai, Hongyu Wu, Ming Chen, Shixiong Liang

**Affiliations:** 1 The Second Affiliated Hospital of Soochow University, Suzhou, People’s Republic of China; 2 Department of Radiation Oncology, Shanghai Pulmonary Hospital,Tongji University School of Medicine, Zhengmin Road, Yangpu District, Shanghai, 200433 People’s Republic of China; 3 Department of the Second Oncology, the First People’s Hospital of Qinzhou, Qinzhou, People’s Republic of China; 4 Department of Radiation Oncology, Zhejiang Cancer Hospital, East Banshan Road, Gongshu District, Hangzhou, People’s Republic of China; 5 Zhejiang Key Laboratory of Radiation Oncology, Hangzhou, 310022 People’s Republic of China

**Keywords:** Interstitial lung abnormalities-radiation pneumonitis-radiotherapy-small-cell lung cancer

## Abstract

**Background:**

Previous studies reported that patients with preexisting radiological interstitial lung abnormalities (ILAs) were more susceptible to developing radiation pneumonitis (RP) after thoracic radiation therapy (TRT). The present study aimed to evaluate the incidence and predictors of RP after TRT in patients with small-cell lung cancer (SCLC) with or without preexisting radiological ILAs.

**Methods:**

A total of 95 consecutive patients with SCLC between January 2015 and December 2015, who were treated with thoracic intensity-modulated radiation therapy at Shanghai Pulmonary Hospital,Tongji University School of Medicine, were analyzed. The diagnosis of ILAs was reviewed by two experienced thoracic radiologists based on the pretreatment high-resolution computed tomography imaging, such as honeycombing, subpleural reticular opacities, ground-glass opacity, and traction bronchiectasis. Univariate and multivariate analyses were used to assess the correlation of clinical factors, preexisting radiological ILAs, and dose-volume histogram-based dosimetric parameters with RP.

**Results:**

Fifteen (15.8%) patients had preexisting radiological ILAs. The incidence of ≥ grade 2 and 3 RP at 1 year was 27.1% and 12.7% in the entire cohort, respectively. Preexisting radiological ILAs were associated with an increased risk of ≥grade 2 RP (50.0% in ILAs + vs 23.3% in ILAs−, *P* = 0.017) and ≥ grade 3 RP (35.8% in ILAs + vs 8.9% in ILAs−, *P* = 0.005) at 1 year. Preexisting radiological ILAs and smoking history (≥40 pack-years of smoking) were significant predictors of ≥grade 3 RP in multivariate analysis (*P* = 0.023 and 0.012, respectively).

**Conclusions:**

Preexisting radiological ILAs and smoking history (≥40 pack-years of smoking) are associated with an increased risk of ≥grade 3 RP after TRT in patients with SCLC.

## Background

Small-cell lung cancer (SCLC) accounts for approximately 10–15% of the total number of cases with lung cancer [1]. The standard treatment for patients with limited-stage SCLC is combined chemotherapy (using platinum-based regimen) and thoracic radiation therapy (TRT) [2]. The outcomes are poor even in the early-stage disease, with a median survival of 19–30 months after curative-intent treatment and 2-year survival of less than 60% [3, 4].

Symptomatic radiation pneumonitis (RP) is a major complication occurring in 15%–40% of the patients with lung cancer receiving concurrent chemoradiotherapy [5]. Previous studies demonstrated that the dose-volume parameters, such as V_20_ (percentage of the lung volume receiving ≥20 Gy), V_5_ (percentage of the lung volume receiving ≥5Gy), mean lung dose (MLD), treatment factors (e.g., sequential/concurrent chemotherapy schedules), tumor factors (e.g., disease location in the lower lung, tumor size), and patient factors (e.g., smoking history, presence of comorbidity), are predictors for developing clinically evident (grades 2–3) radiation pneumonitis. These factors were correlated with RP in patients with lung cancer [5–12].

Interstitial lung abnormalities (ILAs) have a higher incidence in patients with lung cancer compared with the general population [13–15]. Radiographic ILAs were present in 14% of treatment-naïve patients with advanced non-small-cell lung carcinoma (NSCLC) [16]. The widespread use of high-resolution computed tomography (HRCT) in clinical applications has facilitated the detection of ILAs in asymptomatic and undiagnosed individuals [17]. Previous studies reported that patients with preexisting radiological ILAs were more susceptible to developing RP after TRT or stereotactic body radiotherapy (SBRT) [18–21]. Most of these studies were based on patients with NSCLC. The correlation between preexisting radiological ILAs and RP in patients with SCLC is still unclear. Consequently, this single-institution study was conducted to assess all factors as predictors of RP in patients with SCLC based on radiological ILAs, clinical parameters, and dose-volume histogram-based dosimetric parameters.

## Methods

### Patients

From January 2015 to December 2015, 95 consecutive patients diagnosed with SCLC by histology or cytology and treated with TRT were enrolled in the present study. The inclusion criteria were as follows: 1) Karnofsky performance status score ≥ 70, and could endure a definitive RT at a total dose of ≥50 Gy [2.0 Gy/(fraction ⋅ day)]; 2) RT with concurrent or sequential chemotherapy; 3) follow-up time of more than 6 months for patients without RP; 4) thoracic computed tomographic (CT) images available for evaluation before and after RT. The exclusion criteria were as follows: 1) age ≥ 80 years; 2) lobectomy; 3) hypofractionated radiation (> 2.0 Gy/fraction); and 4) SBRT. Informed consent was obtained from all patients. Ethical approval was obtained from the Ethical Review Committee of Shanghai Pulmonary Hospital, Tongji University School of Medicine, China.

### Radiotherapy

All patients underwent a planning CT scan and were immobilized in a supine position with their arms raised in a customized vacuum-lock mold. The simulation CT images were taken in 5-mm increments over the region of interest. Treatment planning was performed with the ADAC Pinnacle TM (Philips Medical Systems) treatment planning system. Treatments were delivered with 6 or 10 MV photons utilizing the thoracic intensity-modulated radiation therapy method on Siemens Artiste (Oncology Care Systems, Siemens Medical Solutions, CA, USA) digital linear accelerator with a multileaf collimator. A gross tumor volume (GTV) was defined as the volume of a primary tumor demonstrated by a CT scan and metastatic lymph nodes that measured ≥1 cm in the short axis. A clinical target volume was typically a 0.5- to 0.8-cm expansion of the GTV, including the primary tumor and the drainage area of metastatic lymph nodes. A planning target volume (PTV) was defined by adding margins at the discretion of radiation oncologists (typically 0.5–1.0 cm for lateral margins and 1.0–2.0 cm for craniocaudal margins, depending on respiratory motion and patient fixation). The planning goal was to deliver the prescription dose to at least 95% of the PTV, while meeting normal tissue constraints. The total dose was ≥60 Gy in limited-stage SCLC and ≥ 50 Gy in extensive-stage SCLC, generally delivered at 2.0 Gy/(fraction ⋅ day, five fractions per week. If the dose of lung exceeds the safety range(V_20_ ≤ 30%, V_5_ ≤ 60%, MLD ≤ 17Gy), we will appropriately reduce the total dose.

### Chemotherapy

The concurrent chemotherapy regimen mainly consisted of cisplatin and etoposide. The second choice for concurrent chemotherapy consisted of carboplatin and etoposide in patients aged > 75 years, with a low-performance status, low renal function (creatinine clearance < 60 mL/min), a syndrome of inappropriate secretion of antidiuretic hormone, or other severe complications. The first-choice chemotherapy regimen consisted of 25 mg/m^2^ of intravenous cisplatin and 70–100 mg/m^2^ of intravenous etoposide on days 1–3. The second-choice chemotherapy regimen consisted of intravenous carboplatin with an area under the curve (AUC) of 5 on day 1 and 70–100 mg/m^2^ of etoposide on days 1–3. The dose of cisplatin, etoposide, or carboplatin in subsequent cycles was reduced by 10–20 mg/m^2^ or AUC of 1 from the planned dose when grade 4 hematologic toxicity or grade 3 nonhematologic adverse events occurred. Chemotherapy was interrupted or changed as a result of prolonged hematologic toxicity, severely elevated creatinine, pulmonary infection, or chemoradiotherapy-induced pneumonitis. Chemotherapy was principally performed in 4–6 cycles every 4 weeks. Patients with older age, stage IV, and poor lung function were considered not suitable for the concurrent chemoradiotherapy; some others were also reluctant to choose this treatment mode due to their poor endurance.

### Radiological ILAs evaluation based on CT

Radiological ILAs assessment was based on pretreatment HRCT imaging with an axial slice thickness of 1 mm in a lung window. Reticular abnormalities, traction bronchiectasis, bilateral independent ground-glass abnormalities, honeycombing, and nonemphysematous cysts were defined as findings indicative of ILAs [17, 21, 22]. Types of ILAs were generally based on history, physical examination, chest HRCT, pulmonary function tests, laboratory tests, bronchoalveolar lavage fluid and lung biopsy. The classification was made according to the American Thoracic Society/European Respiratory Society/Japan Respiratory Society/Latin American Thoracic Association guidelines in 2011 [23]. All CT scans and types of ILAs were evaluated independently by the radiologist and physician specialized in pulmonology.

### Definition of RP grading

According to the Common Terminology Criteria for Adverse Events 4.0, RP was diagnosed based on the clinical symptoms of patients and their radiographic changes on CT scans. RP was graded by several experienced radiation oncologists at the institution according to the following criteria: grade 0, no symptom or radiographic change; grade 1, asymptomatic and radiographic changes only; grade 2, symptomatic but not interfering with the daily life; grade 3, symptomatic but interfering with the daily life, and oxygen is needed; grade 4, life-threatening and ventilator support indicated; and grade 5, death.

### Follow-up

Patients were reevaluated at 1–2 months’ posttreatment and subsequently every 3 or 6 months to check the physical status of patients. Thoracic CT was also performed at each follow-up visit. The endpoint was the incidence of ≥grade 2 RP.

### Statistical analysis

The variables examined were as follows: 1) patient age; 2) gender; 3) tumor stage; 4) tumor location; 5) chemotherapy regimen; 6) percentage forced vital capacity (%FVC), forced expiratory volume in 1 s(FEV1)/FVC; 7) smoking history; 8) ILAs; 9) dosimetric data: total dose, MLD, percentage of lung volume receiving x Gy (Vx), x ≥ 5, 10, 20, 30, 40, and 50, respectively; and 10) GTV and PTV volumes.

The correlation between RP and clinical factors were analyzed using the chi-square test or continuity correction test. The Student t test for linear variables and Fisher exact test for categorical variables were used for univariate analysis. Multivariable logistic regression analysis was performed to evaluate the data for the correlation between clinical factors and dose-volume histogram (DVH) factors with RP. Statistical analysis was performed using SPSS software 22.0 for Mac. A *P* value < 0.05 was considered statistically significant.

## Results

### Patients and treatment characteristics

The characteristics of these patients are presented in Table 1. The present study identified 15 (15.8%) patients with preexisting radiological ILAs. No statistically significant difference was observed in age, gender, smoking history, tumor stage, tumor location, and sequential/concurrent chemotherapy schedules; different chemotherapy regimens compared patients with radiographic ILAs to those without radiographic ILAs. The presence, extent and distribution of ILAs were determined based on chest HRCT criteria in a previous study [19]. None of these patients diagnosed with clinical interstitial lung disease prior to radiotherapy. The characteristics of the patients with preexisting radiological ILAs are presented in Table 2.

**Table 1:** Patient and treatment characteristics for all patients [with ILAs (ILA+) and without ILAs (ILA−)]

Characteristics	All	ILA(+)	ILA(−)	*P* values
	*n* = 95	*n* = 15	*n* = 80	ILA(+) vs ILA(−)
Age (year)				.948
Mean (range)	61 (42–80)	67 (55–80)	60 (42–80)	
Gender				1.000
Male	85	13	72	
Female	10	2	8	
Smoking history (pack-year)				.390
< 40	60	8	52	
≥40	35	7	28	
Tumor stage				.885
IIa	1	0	1	
IIIa	18	3	15	
IIIb	52	9	43	
IV	24	3	21	
Tumor location				.580
Upper or middle lobe	74	13	61	
Lower lobe	21	2	19	
Chemotherapy				.650
Sequential	75	13	62	
Concurrent	20	2	18	
Chemotherapy regimen				.223
Etoposide+cisplatin	61	7	54	
Etoposide+carboplatin	32	7	25	
Other drugs	2	1	1	

*Abbreviations: ILAs* Interstitial lung abnormalities

**Table 2:** Characteristics of the patients with preexisting radiological ILAs

No.	Age (year)/Gender	RT dose (Gy)	Treatment modality	Grade of ILAs	Interstitial change pattern	Involvement of interstitial change	Types of ILAs	Grade of RP
1	80/M	50	RT alone	Slight	Reticular opacities	Right lung	Uncertain	2
2	72/M	50	RT alone	Slight	Honeycombing	Right lower lobe	Uncertain	0
3	78/M	50	RT alone	Moderate	Reticular opacities/ honeycombing	Entire lung	Collagen-vascular diseases	2
4	67/M	60	CCRT	Mild	Reticular opacities	Both lower lobe	Secondary interstitial pneumonia	3
5	71/M	60	RT alone	Slight	Focal reticular opacities	Right lower lobe	Uncertain	0
6	55/M	60	RT alone	Mild	Reticular opacities	Both lower lobe	Uncertain	3
7	64/F	60	RT alone	Mild	Reticular opacities	Both lower lobe	Uncertain	2
8	72/M	60	RT alone	Slight	Focal reticular opacities	Right lower lobe	Uncertain	0
9	59/M	60	RT alone	Slight	Focal reticular opacities	Right lower lobe	Uncertain	0
10	59/M	50	RT alone	Mild	Focal reticular opacities	Both lower lobe	Uncertain	0
11	59/F	60	RT alone	Mild	Reticular opacities	Both lower lobe	Uncertain	0
12	64/M	60	RT alone	Moderate	Reticular opacities/ honeycombing	Entire lung	Secondary interstitial pneumonia	0
13	75/M	60	RT alone	Mild	Reticular opacities/ honeycombing	Both lower lobe	Uncertain	3
14	70/M	60	CCRT	Moderate	Honeycombing/ traction bronchiectasis	Entire lung	IPF	0
15	59/M	64	RT alone	Slight	Focal reticular opacities	Right lower lobe	Uncertain	3

*Abbreviations: ILAs* interstitial lung abnormalities, *RP* radiation pneumonitis, *CCRT* concurrent chemoradiotherapy, *IPF* idiopathic pulmonary fibrosis, *RT* radiation therapy

### RP incidence

**Fig. 1:**
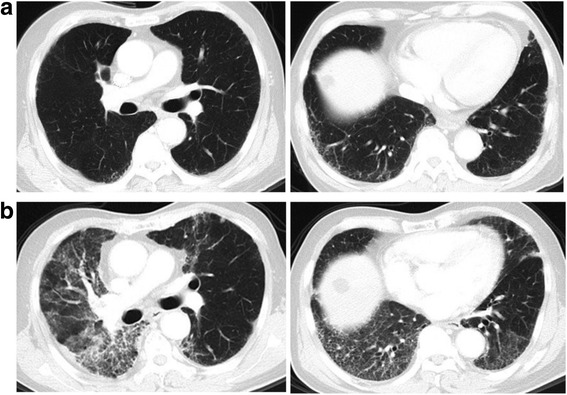
A 75-year-old male with small-cell lung cancer having preexisting radiological ILAs who developed RP beyond radiation field after TRT. **a** Prior to RT, initial HRCT exhibited subpleural reticular opacities at levels of bilateral lower lobes. **b** 60 Gy in 30 fractions was delivered to a pulmonary tumor in the right upper lobe with mediastinum and right hilar lymphadenopathy. Three weeks after completing RT, the patient developed diffuse interstitial infiltration and consolidation extending beyond radiation field

**Fig. 2:**
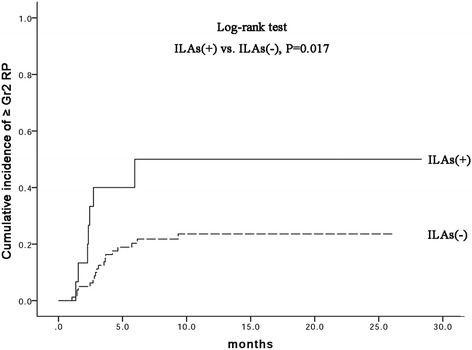
Cumulative incidence of ≥grade 2 RP in patients with and without ILAs

**Fig. 3:**
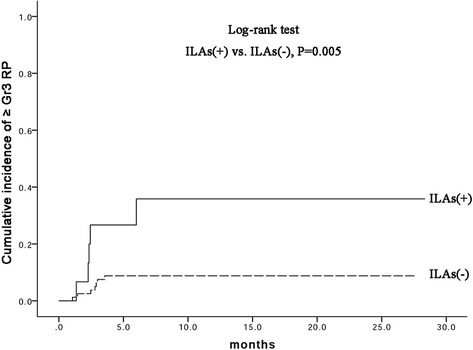
Cumulative incidence of ≥grade 3 RP in patients with and without ILAs

The median follow-up time was 13.0 months (range, 3.4–28.4 months). RP was observed in 33 (34.7%), 13 (13.7%), 12 (12.6%), and 0 (0%) patients with grades 1, 2, 3, and ≥ 4 RP, respectively. Radiotherapy was discontinued in one patient because of RP during radiotherapy. Representative CT images are illustrated in Fig. 1. The cumulative incidence of ≥2 grade at 1 year was 27.1% (95% confidence interval [CI]: 17.3–35.3) for all patients, 50.0% (95% CI: 18.1–75.3) for the radiological ILA(+), and 23.3% (95% CI:13.1–31.9) for the radiological ILA(−). The cumulative incidence of ≥ grade 3 RP at 1 year was 12.7% (95%CI: 5.8–19.4) for all patients, 35.8% (95%CI: 6.3–60.4) for the radiological ILA(+), and 8.9% (95% CI: 2.4–15.1) for the radiological ILA(−). Radiological ILAs were associated with increased risk of ≥ grades 2 and 3 RP (*P* = 0.017 and 0.005, respectively; Figs. 2 and 3).

### Factors associated with RP

Table 3 reveals the correlation between clinical factors and RP. In univariable analysis, radiological ILAs was not found to be a significant factor influencing ≥ grade 2 RP; However, the risk of ≥grade 3 RP was higher in patients with radiological ILAs (*P* = 0.027). Smoking history (≥40 pack-years of smoking) was associated with ≥ grade 3 RP (*P* = 0.012). The correlation between dosimetric factors and RP is presented in Table 4. No correlation was observed between the incidence of RP and dosimetric factors. Any factor significant in univariate analysis was subjected to multivariate analysis using the Cox proportional hazards regression model. Preexisting radiological ILAs and smoking history (≥40 pack-years of smoking) were significant predictors of ≥ grade 3 RP in multivariate analysis (*P* = 0.023 and 0.012, respectively) (Table 5).

**Table 3:** Correlation between clinical factors and RP by univariate analysis

Factors	≥Grade 2 RP			≥Grade 3 RP		
	No. of RPs/Total (%)	*χ*^2^/*F*	*P* value	No. of RPs/Total (%)	*χ*^2^/*F*	*P* value
Gender		.078	1.000		1.616	.442
Male	22/85 (25.9)			12/85 (14.1)		
Female	3/10 (30.0)			0/10 (0)		
Age (year)		.102	.987			1.000
< 70	20/78 (25.6)			10/78 (12.8)	.014	
≥70	5/17 (29.4)			2/17 (11.8)		
Smoking history (pack-year)		.537	.464		8.035	.012
< 40	14/59 (23.7)			3/59 (5.1)		
≥40	11/36 (30.6)			9/36 (25.0)		
Tumor stage		1.494	.684		3.458	.122
IIa	0/ 1 (0.0)			0/1 (0.0)		
IIIa	3/18 (16.7)			0/18 (0.0)		
IIIb	15/52 (28.8)			8/52 (15.4)		
IV	7/24 (29.2)			4/24 (16.7)		
Tumor location		.734	.564		1.513	.391
Upper or middle lobe	21/74 (28.4)			11/74 (14.9)		
Lower lobe	4/21 (19.0)			1/21 (0.5)		
Chemotherapy		.177	.674		.159	.984
Sequential	19/75 (25.3)			10/75 (13.3)		
Concurrent	6/20 (30.0)			2/20 (10.0)		
Total dose (Gy)		.748	.592		1.151	.524
< 54	5/14 (35.7)			3/14 (21.4)		
≥54	20/81 (24.7)			9/81 (11.1)		
FEV_1_/FVC (%)		.106	1.000			1.000
< 70	4/25 (16.0)			2/25 (8.0)	.050	
≥70	6/31 (19.4)			2/31 (6.5)		
FVC (%)		.050	1.000			.747
< 80	4/23 (17.4)			2/23 (8.7)	0.858	
≥80	5/33 (15.2)			1/33 (3.0)		
Radiological ILAs		3.805	.103		6.917	.027
Yes	7/15 (46.7)			5/15 (33.3)		
No	18/80 (22.5)			7/80 (8.8)		

*Abbreviations: FEV*_*1*_ Forced expiratory volume in 1 s, *FVC* forced vital capacity, *ILAs* interstitial lung abnormalities, *RP* radiation pneumonitis

**Table 4:** Correlation between dosimetric factors and RP by univariate analysis

Factors	≥Grade 2 RP			≥Grade 3 RP		
	Without RP	With RP	*P* value	Without RP	With RP	*P* value
GTV volume (mL)	130.2 ± 104.9	122.4 ± 83.9	.758	125.9 ± 101.1	143.6 ± 89.2	.795
PTV volume (mL)	401.3 ± 205.7	395.7 ± 159.0	.484	396.6 ± 196.7	422.1 ± 178.4	.933
MLD (cGy)	1361.6 ± 259.7	1408.6 ± 239.2	.935	1366.0 ± 261.2	1428.9 ± 198.3	.514
*V*_*5*_	48.6 ± 9.4	51.12 ± 9.5	.576	49.0 ± 9.4	50.9 ± 9.8	.730
*V*_*10*_	35.8 ± 7.1	38.2 ± 6.4	.925	36.1 ± 7.0	38.4 ± 6.7	.915
*V*_*20*_	23.7 ± 4.2	25.7 ± 4.2	.929	23.9 ± 4.2	26.7 ± 4.0	.919
*V*_*30*_	16.6 ± 3.8	18.8 ± 4.4	.565	16.8 ± 4.0	19.8 ± 3.8	.782
*V*_*40*_	12.1 ± 3.5	12.9 ± 4.1	.775	12.1 ± 3.6	13.8 ± 3.6	.335
*V*_*50*_	7.7 ± 3.6	7.4 ± 3.3	.534	7.5 ± 3.6	8.0 ± 2.7	.140

*Abbreviations: GTV* Gross tumor volume, *MLD* mean lung dose, *PTV* planning target volume, *RP* radiation pneumonitis

**Table 5:** Correlation between factors and RP by multivariate analysis

Factors	B	S.E	Wald	df	*P*	EXP (B)	95% EXP(B)	
							Lower	Upper
Radiological ILAs	−1.652	.728	5.154	1	.023	.192	.046	.789
Smoking history (≥40 pack-years of smoking)	−1.828	.731	6.252	1	.012	.161	.038	.674

*Abbreviations: ILAs* interstitial lung abnormalities, *RP* radiation pneumonitis

## Discussion

Previous studies demonstrated that the dose-volume parameters and treatment factors are predictors for developing RP after TRT in patients with SCLC. In addition, the patient factors are also closely related to the incidences of RP. Previous studies reported that patients with preexisting radiological ILAs were more susceptible to developing severe, extensive RP after TRT or SBRT in lung tumor [18–21]. Severe ILAs was regarded as a relative contraindication in the clinical guidelines for SBRT published by the Japanese Society for Therapeutic Radiation and Oncology [19]. Most of these studies were based on patients with NSCLC. The correlation between preexisting ILAs and RP in patients with SCLC is still not clear. The present study examined the clinical and dosimetric factors as predictors of RP and evaluated the correlation between ILAs and RP. The findings revealed that the presence of preexisting radiological ILAs was also associated with increased risk of ≥ grade 3 RP. This was a new finding for the radiation oncologists because few studies focused on the correlation between radiological ILAs in preradiation therapy HRCT and RP in patients with SCLC. These raised the important pointers to preexisting radiological ILAs in lung cancer, increasing the chances of an “exacerbation,” particularly with radiation therapy. The patients with SCLC having preexisting radiological ILAs must be carefully watched during chemoradiotherapy and closely monitored following TRT.

TRT is an important component of treatments for lung cancer, especially for limited-stage SCLC. However, the optimal dose and fractionation for TRT in limited-stage SCLC remain controversial; a hyperfractionated regimen (45 Gy in 30 fractions delivered as 1.5-Gy fractions twice daily, BID group) or a conventionally fractionated regimen (60–70 Gy in 30–35 fractions delivered as 2.0-Gy fractions once daily, QD group) has been used in routine clinical practice. Pneumonitis and dermatitis were more common in the QD group, and esophagitis was more common in the BID group [24, 25]. Possible differences in toxicities depending on RT regimen may be worth further investigation. Tsujino et al. reported that the incidence of RP was lower in the BID group than in the QD group after treatment. The incidence of ≥grade 2 RP increased with increases in V_20_, and the DVH parameters (especially V_20_) were used to predict symptomatic RP in patients undergoing BID TRT [8]. Data on the clinical and dosimetric parameters that predict RP in patients with SCLC treated with QD TRT are limited. Consequently, this retrospective analysis was conducted to contribute data on this series. No correlation was observed between the incidence of RP and dosimetric factors in the present study. The main reason for this lies in we strictly controls the limits of V_20_, V_5_ and MLD. In designing a plan, we must ensure the safety of lung firstly. If the dose of lung exceeds the safety range, we will appropriately reduce the total dose.

Till now, the influence of smoking history on RP risk is unclear. The smoking history (≥40 pack-years of smoking) was another significant clinical predictor of ≥grade 3 RP in the present study, consistent with a previous study [12]. In an analysis of 297 patients receiving SBRT for a lung tumor, pack-years of smoking was a significant predictor of RP in the multivariable analysis [10].

Concurrent chemoradiotherapy (CCRT) is recommended as a standard treatment for limited-stage SCLC. The patients with severe pulmonary complications, older age, were considered unfit to undergo concurrent chemotherapy received sequential chemoradiation in this study. Some of the patients was given sequential chemoradiation because of an advanced stage. There was no significant difference in the concurrent and sequential groups in this study. Only one retrospective study recently concluded that pulmonary fibrosis was observed significantly more often after concurrent than sequential chemoradiation in patients with limited-stage SCLC [26]. But the dose fractionation schedules were different from ours in a portion of patients. This result still needs more research to confirm the conclusion.

The clinical factors including larger gross internal tumor volume, PTV, and location of tumor were predictors of RP [6, 10]. In the present study, neither the location of tumor nor the size of tumor (GTV) had any impact on the incidence of ≥grade 2 or 3 RP. This was because the majority of SCLCs arose centrally; primary tumors located in the peripheral or lower lobes were rare. Moreover, patients with SCLC were more sensitive to chemotherapy than those with NSCLC; the irradiation field was smaller after induction chemotherapy in patients who had a partial response in GTV. The patients with a V_20_ ≥ 35% might benefit from induction chemotherapy due to an estimated reduction of RP [27].

This study had some limitations. First of all, as a retrospective study and a small sample size with short inclusion were analyzed, this study has the possibility of selection bias and confounding factors. In addition, the diagnosis of radiological ILAs was based on pretreatment CT imaging and evaluated by two experienced thoracic radiologists; the differences in subjective judgment may vary. A larger, preferable prospective data set is needed to confirm these conclusions.

## Conclusions

Preexisting radiological ILAs and smoking history (≥40 pack-years of smoking) are clinical risk factors for severe RP in patients with SCLC. Prospective studies are needed to validate these factors.

## Abbreviation

AUC: Area under the curve
CCRT: Concurrent chemoradiotherapy
CI: Confidence interval
CT: Computed tomography
DVH: Dose–volume histogram
FEV_1_: Forced expiratory volume in 1 s
FVC: Forced vital capacity
HRCT: High-resolution computed tomography
ILAs: Interstitial lung abnormalities
MLD: Mean lung dose
NSCLC: Non-small-cell lung carcinoma
PE: Pulmonary emphysema
PTV: Planning target volume
RP: Radiation pneumonitis
SBRT: stereotactic body radiation therapy
SCLC: Small-cell lung cancer
TRT: Thoracic radiation therapy
V_10_: Percentage of lung volume receiving 10 Gy
V_20_: Percentage of the lung volume receiving ≥20 Gy
V_30_: Percentage of lung volume receiving 30 Gy
V_40_: Percentage of lung volume receiving 40 Gy
V_5_: Percentage of the lung volume receiving ≥5 Gy
V_50_: Percentage of lung volume receiving 50 Gy
